# Participatory evaluation of municipal obesity prevention clubs in Tehran city: Strengths, challenges, and future direction

**DOI:** 10.3389/fpubh.2023.1055210

**Published:** 2023-02-16

**Authors:** Sareh Edalati, Nasrin Omidvar, Amirhossein Takian, Farzaneh Rasam, Delaram Ghodsi, Reza Majdzadeh

**Affiliations:** ^1^Department of Community Nutrition, National Nutrition and Food Technology Research Institute, Faculty of Nutrition Sciences and Food Technology, Shahid Beheshti University of Medical Sciences, Tehran, Iran; ^2^Department of Global Health and Public Policy, School of Public Health, Tehran University of Medical Sciences, Tehran, Iran; ^3^Department of Health Management, Policy and Economics, School of Public Health, Tehran University of Medical Sciences, Tehran, Iran; ^4^Health Equity Research Centre, Tehran University of Medical Sciences, Tehran, Iran; ^5^Department of Social and Cultural Affairs of Tehran Municipality, Health Department, The Tehran Municipality's Organization, 15th District, Tehran, Iran; ^6^Department of Nutrition Research, National Nutrition and Food Technology Research Institute, Faculty of Nutrition Sciences and Food Technology, Shahid Beheshti University of Medical Sciences, Tehran, Iran; ^7^Community-Based Participatory Research Center, Tehran University of Medical Sciences, Tehran, Iran; ^8^Knowledge Utilization Research Center, Tehran University of Medical Sciences, Tehran, Iran; ^9^School of Public Health, Tehran University of Medical Sciences, Tehran, Iran

**Keywords:** obesity, obesity prevention, community-based program, evaluation, participatory evaluation, program evaluation, community-based organization (CBO)

## Abstract

**Background and aim:**

Community-based initiatives are important for obesity prevention. This study aimed to evaluate the activities of municipal obesity prevention clubs (OBCs) in Tehran, Iran, using a participatory approach.

**Methods:**

The evaluation team was formed, and the members identified the OBC's strengths, and challenges and provided suggestions for change through a participatory workshop, observation, focus group discussions, reviewing relevant documents (*n* = 97), and 35 interviews with involved stakeholders. The MAXQDA software was used for data analysis.

**Results:**

An empowerment training program for volunteers was identified as one of the strengths of OBCs. Despite the obesity prevention efforts of OBCs through public exercise sessions, healthy food festivals, and educational sessions, several challenges were identified that hinder participation in OBCs. These challenges included poor marketing strategies, poor training approaches in participatory planning, insufficient motivational support for volunteers, low perceived recognition of volunteers by the community, volunteers' low food and nutrition literacy, poor educational services in the communities, and limited funding for health promotion activities.

**Conclusion:**

Weaknesses in all stages of community participation, including information, consultation, collaboration, and empowerment, in OBCs were detected. Facilitating a more enabling environment for informing and involving citizens, expanding neighborhood social capital, and involving health volunteers, academia, and all potential governmental sectors to collaborate for obesity prevention is recommended.

## Introduction

Non-communicable diseases (NCDs) are a major burden worldwide, particularly in low- and middle-income countries (LMICs), i.e., the Eastern Mediterranean region (EMR). In the meantime, overweight and obesity, as one of the main risk factors for NCDs (1), are rapidly on the rise in the region. In Iran, the last national CASPIAN-V survey of 2015 and the stepwise approach to NCDs surveillance (STEPs) survey of 2016 revealed the cumulative prevalence of overweight and obesity at 20.9 and 59.3% among children (7–18 years of age) (2) and adults (3), respectively. High body mass index (BMI) is attributed to 18.8% (95% UI: 13.2–24.7) of deaths and 12.9% (95% UI: 9.3–16.7) of DALYs related to NCDs in Iran (3). Therefore, in line with the spell out in the first use global action plan for prevention and control of NCDs (4), the Ministry of Health and Medical Education (MoHME) of the country defined halting the rise of obesity prevalence by the year 2030 as a national target in the national action plan for prevention and control of NCDs (5). Achieving this goal will require a social approach at all governmental levels (5).

The World Health Organization (WHO) framework on community participation for health advocates strengthening the participation of local communities through different stages, including information, consultation, collaboration, and empowerment, as a vital approach for disease prevention and health promotion (6, 7). Similarly, WHO-EMR advocates involving citizens to address different urban health issues and their socio-ecological determinants through community-based initiatives or voluntary work (8–11). Several EMR countries have attempted some related initiatives in this regard (6, 11, 12). In Iran, the MoHME has launched various community-based health programs, including the primary health care (PHC) network, women's health volunteer program, student-peer education, and the healthy city program (13, 14). In addition, some non-health sector organizations, e.g., local governments and municipalities, have also contributed to health promotion programs, e.g., community-based (CB) “health clubs” affiliated with the Tehran municipality (14). These community-based health clubs are non-profit organizations based on the voluntary work of citizens who are interested in health issues and get involved in planning and implementing programs to improve citizens' health. These are different clubs, including obesity prevention, blood donation, the elderly, youth, physicians, smoking cessation, mother and child, environment, mosque, disabled people, and diabetes prevention. Volunteers are encouraged to identify and involve related stakeholders from governmental and non-governmental organizations (NGOs) to plan and implement health promotion initiatives through the clubs (15).

The obesity prevention clubs (OBCs) aim to initiate neighborhood obesity prevention activities through the collaboration of volunteer citizens and health-house programs (14). The main aims of obesity prevention clubs are to empower citizens to practice a healthy lifestyle, provide educational and counseling services to overweight and obese citizens to achieve and maintain a healthy weight, and engage citizens in social network support groups to promote activities to improve their health (15). Ever since these clubs began in 2012, their performance has not been evaluated. This study aimed to evaluate community-based activities and the performance of OBCs for obesity prevention in Tehran city, using a participatory approach.

## Materials and methods

In this cross-sectional study, a participatory approach was used to evaluate the activities of OBCs within CB health organizations. Participatory research (PR) is an approach that prioritizes co-constructing research through involvement and collaboration between researchers, organizational representatives, and community members in all phases of the research process (16). PR values the engagement of those who are beneficiaries, users, and stakeholders of the research in the research process rather than including them only as subjects of the research (17). Community-friendly and participatory approaches to evaluation are highly recommended and applied in evaluating community activities (18, 19) and are considered a form of “citizen science”, focusing on the use of public participation to collect and share data with scientists and empower citizens to identify their own needs and concerns within a community (20, 21).

To set up the evaluation team, we contacted different stakeholders related to obesity-prevention clubs. The inclusion criteria for participants of the evaluation team were having experience and active involvement in obesity prevention clubs for at least 4–5 years, as well as being able and showing interest to participate. The municipality's leaders and community members were contacted through phone calls. The final evaluation team included community citizens, i.e., volunteers in the program, as well as health managers at district and city levels.

**Figure 1:**
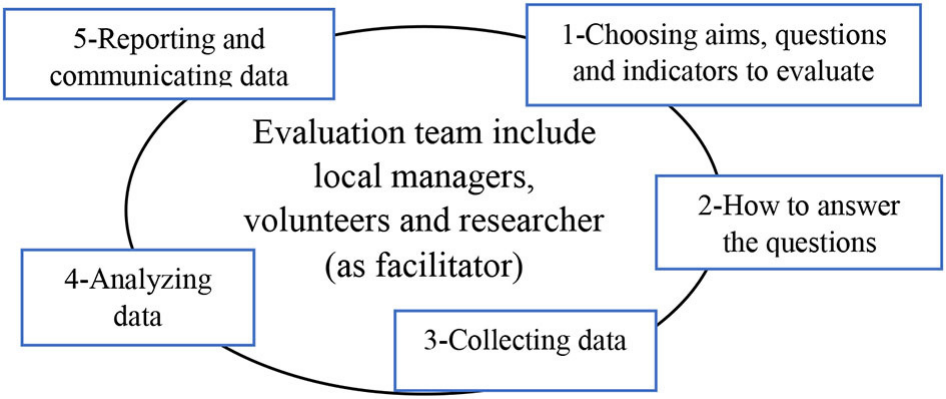
Steps in participatory approach to evaluation.

**Figure 2:**
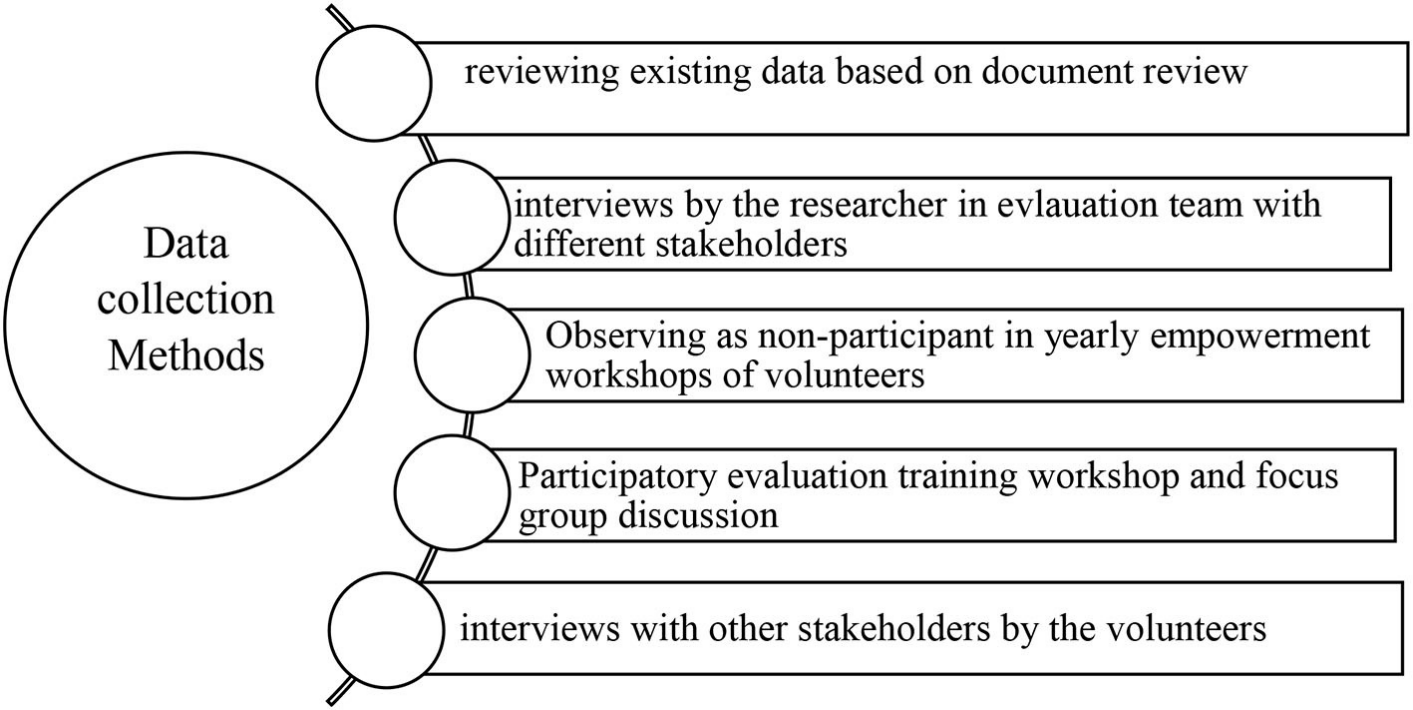
Data collection methods used in evaluation of local obesity-prevention clubs in Tehran city.

It was also assured that we share the same language through different phases of evaluation, including defining the aims and main questions, the data collection methodology, and how to involve stakeholders in different phases of evaluation (Figure 1). Several meetings were held to reach a consensus on the evaluation process and to establish our team's contact with the district-level manager in the health department of the Tehran municipality (FR). The main aims of the evaluation team were set jointly, which included the following: (1) to describe a timeline to identify the activities and programs of obesity prevention clubs (history and its evolution); (2) to identify the strengths and challenges of the activities and programs of the obesity prevention clubs; and (3) to present suggestions to improve the activities and programs of obesity prevention clubs. The evaluation team performed data collection from November 2019 to September 2021, using five methods (Figure 2), as follows:

### Review of the existing documents

Key Internet websites, including Tehran Urban Research and Planning Center (https://rpc.tehran.ir/), social and cultural affairs deputy of Tehran Municipality (https://farhangi.tehran.ir/), national news agencies, Google, Google scholar, and Scopus, were searched for any article, guidelines, documents, or reports related to the activities of municipal OBCs with no limit, using key relevant search terms, including obesity prevention clubs, community-based (CB) health clubs, community-based health organizations, Tehran municipality, and obesity prevention, in English and Persian language. Minutes, guidelines, books, and documents were also obtained by the participatory evaluation team *via* face-to-face interviews and meeting sessions.

### Semistructured interviews with the program stakeholders

Interviews with informed stakeholders, involved in the design and implementation of the program, were performed by one of the research team members (SE), using a tailored interview guide (Supplementary File 1). We used both purposeful and snowball sampling. The interviewer also asked probing or follow-up questions, if needed. All interviews were conducted face-to-face in the neighborhood departments, except one, which was done *via* phone call. Each interview lasted for 20–60 min. Data reached saturation at the 20th interview when the interview did not yield any new or additional information and concepts despite the varying backgrounds of participants; however, four additional interviews were done to ensure no new information could be generated. A total of 44 individuals participated in this study.

### Observing annual empowerment and training workshops for volunteers

A 1-day physical empowerment workshop (8 h) was held for volunteers in a health house on February 2020. A team member (SE) joined and observed the annual empowerment workshop of volunteers to track the content and follow-up acts. The main goal of the workshop was to educate volunteers about facilitation techniques and get them familiar with community engagement methods. In this workshop, out of 22 invited volunteers, 18 attended. The first part of the workshop included brainstorming and discussion to reflect on volunteers' experiences regarding the challenges they face in obesity prevention clubs. The session was recorded, and notes were taken for further analysis through the triangulation of data. The facilitator described the definitions of the group and community-based organizations, as well as participation levels and facilitation techniques. Afterward, volunteers got familiar with problem/solution tree analysis for obesity in the district they came from. The facilitator led the participants in identifying the causes and consequences of each problem in paired groups. The participants were encouraged to apply what they have learned when implementing the obesity prevention clubs' activities.

### Participatory evaluation training workshop and focus group discussion with local volunteers

A 1-day participatory workshop was held on September 2020 in a municipal community center of “Aboozar” in district 14 of Tehran. The workshop content and methodology were finalized through consultations with the evaluation team while getting insight from reviewing participatory evaluation methodologies (21–25) and adaptations for remote work (26) due to the COVID-19 pandemic. Nine participants were invited and joined the workshop. Each participant received a complementary health package (one mask and an alcohol-based hand sanitizers) and travel expenses when leaving. The participatory workshop included two parts. The first part included ice breaking (14 min), brainstorming (8 min), followed by the training session (50 min). Then, after a break, in the second phase of the workshop, participants were encouraged to apply what they had learned in the first section to evaluate their activities through a focus group discussion session (90 min). The details of the steps followed in the workshop are as follows.

#### Step 1-Ice breaking (14 min)

The chairs were arranged in a *U* shape to increase eye and verbal contact between the participants. An ice-breaking technique was used to ease group contributions and help participants get to know each other. The facilitator asked the participants to break into pairs and introduce themselves to each other. Then, each participant introduced her partner to the rest of the group.

#### Step 2-Brainstorming (8 min)

The facilitator asked the participants to express the first words or ideas that came to their mind when they heard “participatory evaluation”. She wrote and listed the participants' views on large sheets of butcher papers placed on a whiteboard without any judgment and in a neutral manner.

#### Step 3-Training (50 min)

To enhance participants' evaluation capacity and knowledge, SE simply explained participatory evaluation by paraphrasing what they had said and summarizing key ideas about participatory evaluation. They were also given a guide to participatory evaluation. This training about the benefits of participatory evaluation and documentation in their organizations, as well as how to implement participatory evaluation participants also encouraged them to share their stories if they had previously done participatory evaluation during their previous activity.

#### Step 4-Interactive focus group discussion session (90 min)

A focus group discussion was held to understand the stakeholders' views regarding their own and/or health center local managers' activities, their strengths and challenges, and their suggestions after reviewing the main objectives of the program. The main questions asked were as follows. “how well the OBC have achieved their goals?” “Are they successful?” “What do you perceive as the best practices and strengths of the program which you are proud of?,” and “What challenges you faced?” All the shared ideas were written down on large sheets of butcher paper placed on a whiteboard. The evaluation team summarized their answers into meaningful sentences after member checking.

The discussion flowed with minimal prompting and a neutral tone of voice without exhibiting either positive or negative bias to anything mentioned by the respondents to prevent dominance and include everyone in the discussion. When a participant was unclear, the facilitator clarified, probed, and gathered details about their shared ideas through paraphrasing, e.g., “Are you saying…?” or “Am I understanding you to mean…?” to ensure that the information was understood and to improve the depth of understanding. When the participants appeared to be contributing very unequally, with certain individuals dominating and others not being heard at all, the “managing talk” play technique adopted from the UNICEF manual on participatory events (27) was applied. Each participant got three pieces of beans as three chances to speak in front of the group. After they had returned all their beans, they could no longer speak up.

##### Exploring volunteers' suggestions and scoring them (50 min)

Participants were asked the following prompt question. “What do you suggest to overcome the challenges identified to improve the obesity-prevention club activities in the future?” Their shared suggestions to overcome the challenges were written on butcher papers placed on a whiteboard. Then, the whiteboard turned around and faced the wall to ensure privacy considerations. Each participant scored the suggestions, one-by-one, by adding smiley face emoji stickers of different colors beside each suggestion. The red, yellow, blue, and green stickers represented 1, 2, 3, and 4 agreement scores, respectively. The workshop was recorded with the consent of the evaluation team for further analysis. Also, photographs were taken from the butcher papers for documentation and further analysis.

### Step 5-Interviews with other stakeholders by community volunteers

Coinciding with the COVID-19 pandemic and its limitations and ensuring the health of all stakeholders participated and enhance participation, we made adaptations and adjustments to the study protocol (26). For the self-evaluation of the program, a virtual online WhatsApp group was initiated. All nine participants who had attended the evaluation workshop joined the WhatsApp group and were asked to interview other stakeholders by focusing on the following two main questions: What are the strengths and challenges of obesity prevention clubs? and What are their suggestions to improve these clubs? Out of nine participants, seven (participation rate, 77%) collaborated in interviewing with other stakeholders (*n* = 11). They presented and invited other citizens involved in the program, interviewed them about the program's strengths and challenges, and requested their suggestions to improve the program.

### Data analysis

A thematic content analysis was applied to analyze data from document reviews, transcribed semistructured interviews, and focus group discussions within the empowerment and participatory workshop. The results (codes, subthemes, and themes) were based on the triangulation of different sources of data, which had been collected through a variety of methods, including participatory workshops, observation, focus group discussion, reviewing relevant documents, and stakeholder interviews. SE read the transcriptions several times carefully and word-by-word in order to capture key thoughts and concepts in open coding. Open codes were initially identified, and then similar codes were combined to form themes and sub-themes. The MAXQDA software (version 18, Berlin, Germany) was used to manage data analysis.

### Data trustworthiness

Purposive and snowball sampling provided diverse views and allowed transferability. To improve data consistency, another team member (DG) independently recoded and reanalyzed 40% of the interview transcriptions (randomly selected from different sectors). The generated themes and sub-themes and potential differences were then compared and discussed until we reached a consensus on the final codes and themes. Any discrepancies were resolved through discussion with another team member (NO). The final set of themes and sub-themes were discussed and agreed on with the wider evaluation team. The consolidated criteria for reporting qualitative research (COREQ) (28) were used to guide data analysis.

### Reporting and communicating the evaluation process

The Skyroom platform was used for holding two virtual meetings with the evaluation team to finalize and reach a consensus regarding data analysis and interpretation. Different ways to communicate data were also discussed.

## Results

### Participant characteristics

The main characteristics of the participants who took part in each qualitative data collection method are presented in Table 1. The participants of the participatory workshop and focus group discussion were the same, although those in the semistructured interviews were not. Interviewees were local managers (*n* = 12), district and neighborhood volunteers (*n* = 21), and nutrition consultants who worked in the clubs (*n* = 2) (Table 1).

**Table 1:** Main characteristics of individuals participated in different data collection methods.

**Data collection method**			**Characteristics of participants**						
			**Total** **(number** = **44)**	**Gender**		**Education level**			
				**Female** ***n*** **(%)**	**Male** ***n*** **(%)**	**High school diploma** ***n*** **(%)**	**Associated degree** ***n*** **(%)**	**Bachelor** ***n*** **(%)**	**Higher degree** ***n*** **(%)**
1- Focus group discussion with volunteers			9	9 (100)	0 (0)	3 (33.3)	2 (22.2)	3 (33.3)	1 (11.1)
2 Interview sessions by the evaluation team	First author (researcher)	Local managers	8	7	1	0	0	6 (75)	2 (25)
		District and local volunteers	14	14	0	7 (53.8%)	2 (15.3)	3 (23.7)	2 (14.2%)
		Nutrition consultants	2	2	0	0	0	0	2 (100)
	Volunteers	Local managers	4	4 (100)	0	0	0	4 (100)	0
		District and local volunteers	7	7 (100)	0	2 (28.5)	3 (42.8)	2 (28.5)	0

### Document review

Overall, 97 documents were identified and reviewed for analysis. Characteristics of the reviewed documents are presented in Supplementary File 2 and Table 1.

### Defining participatory evaluation

**Figure 3:**
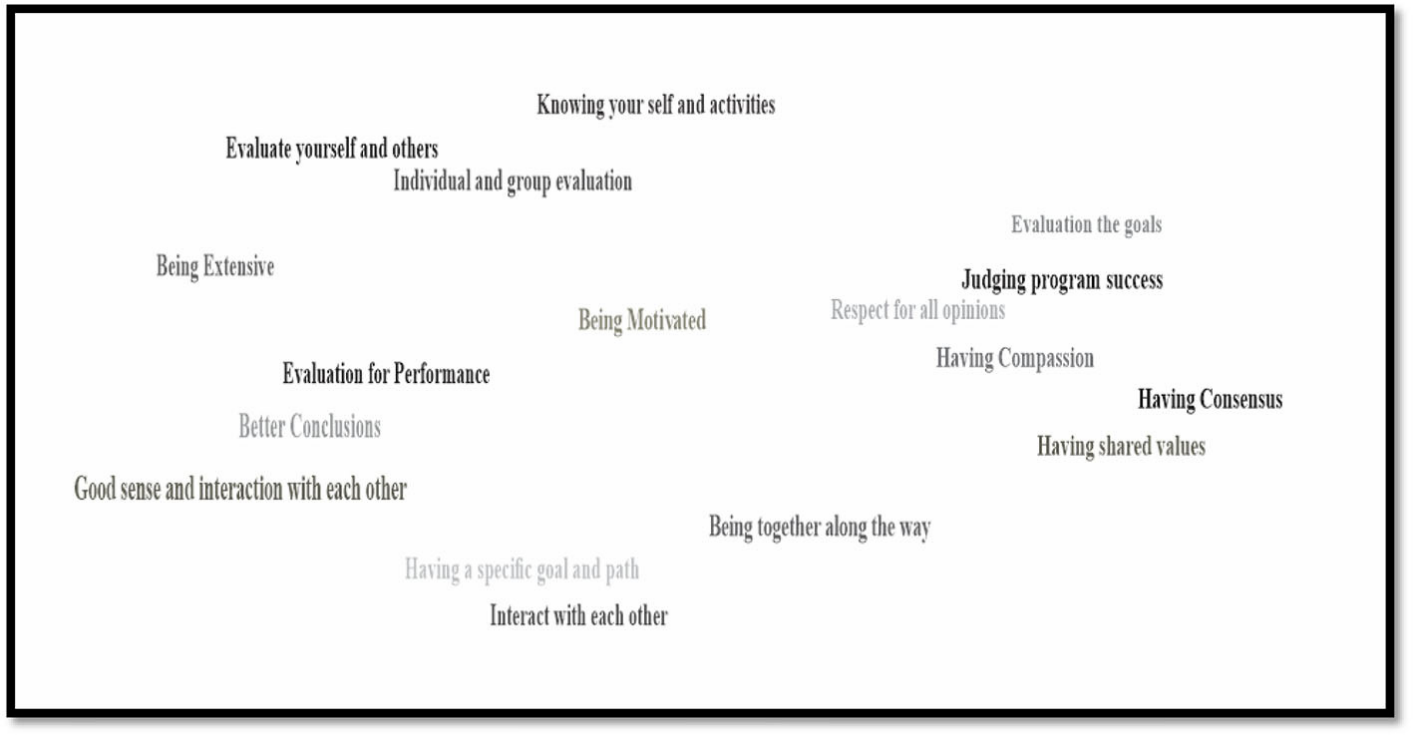
The word cloud about participatory evaluation and its characteristics developed through brainstorming by volunteer citizens.

The main themes extracted during the workshop from brainstorming notes about participatory evaluation and its meanings are presented in Figure 3, as a word cloud.

### History and evolution of obesity prevention clubs

**Figure 4:**
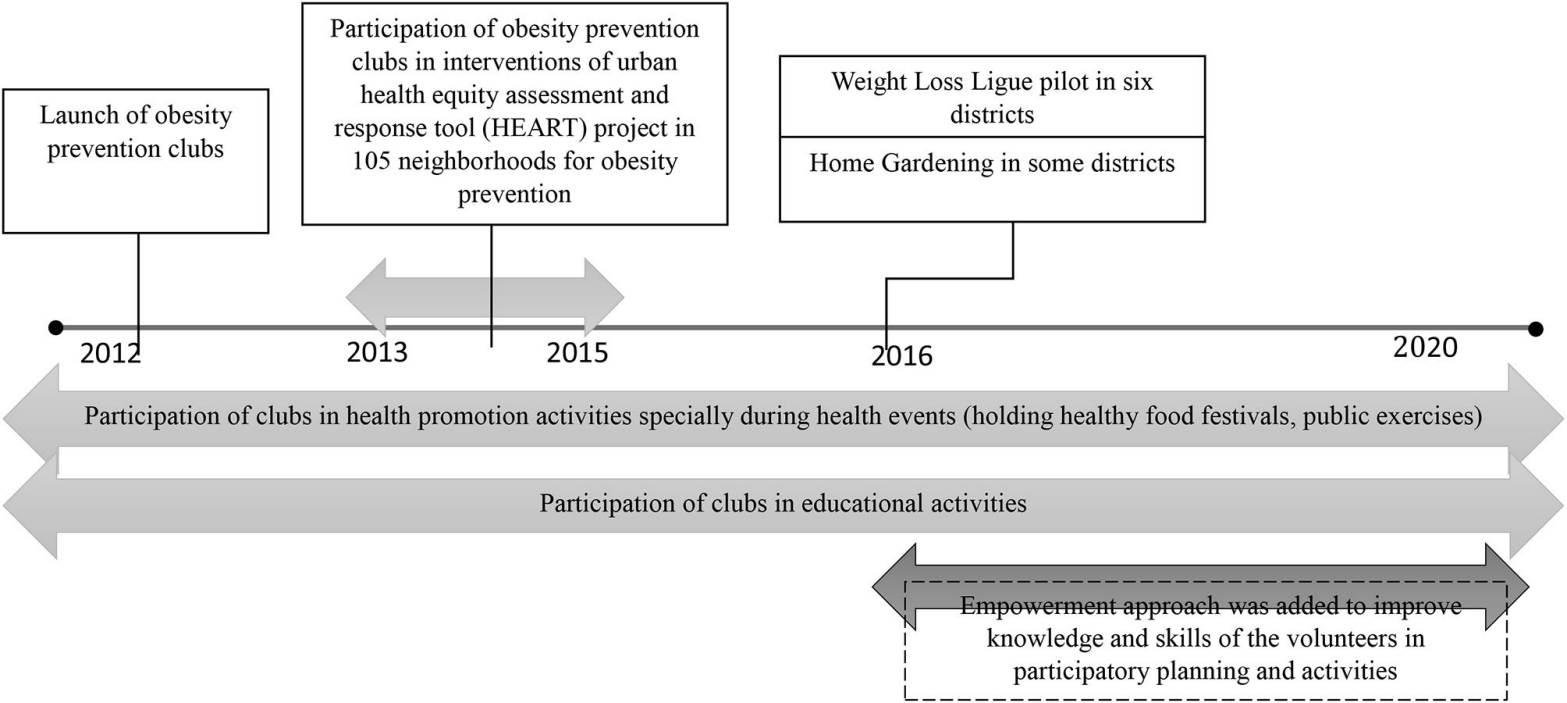
Timeline of activities within local obesity prevention clubs in Tehran city.

A participatory timeline method (29) was applied to overview the main activities of the OBC over time. An initial timeline was shown to the community members through virtual meetings, and they were asked to place or correct the overall history and its milestones. The final timeline is summarized in Figure 4. As shown, OBCs were developed in 2012. Their main activities included communicating with local nutritionists and physicians to hold nutrition education sessions, as well as designing healthy food festivals and group sessions of physical activity, mainly during the health events. Reviewing documents and interviews showed that activities within the clubs evolved from a biomedical approach to healthcare to a social empowerment approach, emphasizing on improving volunteers' skills for facilitating activities and participatory programming.

In the first stage, the activities were mainly focused on the physical and biological aspects of obesity that were limited to screening its related risk factors, including blood pressure and blood glucose (fast testing) measurement by local volunteer leaders at the health clubs. Gradually, these screening activities were stopped, local volunteers got more involved in community-based initiatives, and a social model of healthcare was promoted which involved conducting small projects. To do this, during short courses provided since 2016, local volunteer leaders were trained in order to improve their knowledge and skills about participatory planning and activities. Also in the same year, a workshop was held to educate volunteers about teamwork and sharing activities.

In 2019, a 3-day summer school for community-based health clubs was held to improve the knowledge and skills of volunteers about participatory planning. The main topics included in this training were as follows: clubs and their role in local development; how to improve participation, teamwork, capacity building, clubs' rights and developing a vision, participatory planning, writing SMART objectives, ABCD-based activities, documentation, and participatory evaluation; and critical assessment of OBC. Also, another workshop was held on February 2020 for district volunteer leaders. In this workshop, volunteers received training about group formation, participation and its different types, facilitation and participation techniques, and how to define problems and solutions through a problem tree.

### Perceived strengths of obesity prevention club activities

According to stakeholder's comments, citizens' participation in health activities at the neighborhood level, improving citizens' knowledge about healthy eating, developing infrastructure within the clubs, hosting empowerment training workshops for volunteers since 2016, demonstrating the interest and loyalty of volunteers, and engaging obesity prevention activities during the pandemic were the most significant activities of the OBCs, which they were all proud of.

Major health activities that OBCs were involved in included home gardening, interventions related to the urban HEART program, the weight reduction league, and collaboration with research projects. Incorporating health promotion activities at the neighborhood level in different programs and projects was the main activity they were proud of; as one of the volunteers' leaders remembered participating in developing a home gardening program in her district:

“*We had a home gardening program. We aimed to teach citizens to grow vegetables and use them in their own home. It was in District 22, two years ago. I have no knowledge like the agricultural expert, this is not my specialty. Therefore, we contacted an agriculture expert from the plant and flower clinic. We invited the members of the obesity prevention clubs and agricultural experts trained them. The slogan of the world food program in that year was” from farm to your table”. After the training session, one of the members invited me to her home. She had planted mint and used it for her family. Another member invited me to her house. She had planted beans, eggplant and peppers.... She said, it was beautiful to spend time with them every day and used it as a source of fiber in the family meals. They liked it very much. This was an interesting project I remember” (a volunteer, interview)*.

Involving OBCs in implementing interventions related to the urban HEART program in 105 neighborhoods was another activity they highlighted and considered significant. An example is what happened in the “Doolat-Abad” neighborhood (in district 20) where healthy “Falafel”, a southern Iran dish, in the neighborhood fastfood stores was promoted after consulting with a nutritionist. The revised recipe included more vegetables and using healthy oil. In the preparation of Falafels, it was served with doogh (a yogurt drink) as a drink instead of cola.

The inclusion of empowerment training workshops since 2016 was the other strength mentioned by the stakeholders. These annual workshops aimed to empower people in participatory planning.

### Perceived challenges of the clubs in obesity prevention activities

Table 2 presents participant's perceptions regarding the main challenges of OBCs. Poor knowledge of citizens about health-oriented services of the municipality and OBC was among the challenges identified. Some examples of the stated challenges are as follows:

“*The municipality is not known for its health activities. I was introducing the health services of municipality to my neighbor and explained to her about low price pools as a subset of the municipality. She didn't believe. A woman came and said I have no money. The doctor told me to go to the pool for weight loss and resolving obesity associated problems. I was able to get 40% discount for her, which made her very happy; people do not know about these facilities.” (A health club volunteer, interview)*

**Table 2:** Themes and sub-themes extracted regarding perceived challenges of stakeholders for implementing obesity prevention clubs' activities.

	**Themes (*n* = 13)**	**Subthemes (*n* = 40)**
1	Low desire of citizens for membership in obesity prevention clubs	• Insufficient and poor knowledge of citizens about health-oriented services of the municipality • Time limitation • Lack of financial power to attend educational classes • Unattractiveness of services provided elimination of facilities for using free sports services or discounts for members
2	Poor marketing strategies for the clubs	• Insufficient advertising of facilities • Decreased activities of “Salamat-yar” • Ads disproportionate to the local context of the neighborhood • Not using the capacity of the media and limited recruitment method to the neighborhood houses.
3	Poor participatory skill development in volunteers	• Inadequate empowerment of volunteers in soft skills required for collaborative activities • Poor experience exchange between empowered and less empowered volunteers • Top-down planning which has resulted in dependence of the obesity prevention clubs to the health department • Unclear definition of participatory action in the executive instructions • Inadequate support and facilitation of health managers for volunteers for participatory planning • Inadequate context needed for participation in organizations • Not having identity and credibility which hinders collaborating with other organizations.
4	Poor empowerment training courses for volunteers for action and participatory planning	• Weaknesses in skills needed for participatory planning • Not considering specialized training
5	Insufficient motivational support (e.g,. financial incentives) for volunteers	• Insufficient incentives programs for volunteers • Not paying volunteer's traveling costs for attending meetings in some health districts
6	Weaknesses in the process of educational services	• Insufficient human capacities to provide free or low-cost training services • Poor selection criteria of instructors, Weak communication skills in training by some invited nutritionists • Contradiction of modern and traditional nutrition information offered through educational sessions lack or weakness of effectiveness evaluation of the programs
7	Weaknesses in exercise and sport activities	• Limited physical infra-structure for public exercises • Social limitations for public exercising
8	Weaknesses in virtual activities of health clubs (after pandemic)	• No meaningful and goal-oriented management for infodemic problem • Sharing non-scientific messages and gossiping
9	Limited funding for health promotion activities	Low financial support for health promotion activities
10	Poor collaboration or motivation of volunteers	• Busy personal life • Low motivation due to low support • Identifying problems
11	Low food and nutrition literacy of volunteers	• Low knowledge of volunteers regarding nutritional issues • Low media food and nutrition literacy of volunteers
12	Instability of the programs	Instability of programs due to manager changes and social issues
13	Poor participatory documentation and evaluation	Poor system for participatory documentation and evaluation

Regarding inadequate empowerment of volunteers in the soft skills required for collaborative activities, a local manager said:

“*We still have a lot of room to empower our volunteers. We did poorly in this regard. For us, as managers at the health house, several courses were held to learn facilitating activities. After ten years, I know the principles of facilitation. I know how to work with people. We need more training for the volunteers in this regard. Many times, volunteers come to me to coordinate with an organization, e.g., to initiate an activity or to hold a nutrition education session. I ask them to contact a university themselves. I want them to learn bargaining. Or, I ask them to write a letter to involve those they wished to collaborate on in this regard. “Now, they can write a letter. This is very important.” (local manager, interview)*

Most of the participants believed that one of the weaknesses of the clubs is poor accountability and credibility. The OBCs have no credentials; they are not even recognized in the municipality structure, and this is why they do not have any identity to collaborate with other organizations. As one of the local managers stated:

“*Even inside the municipality department, we don't give a verdict to volunteers because of legal problems. It causes ownership and insurance issues. (a local manager, interview)”*

More attention to theoretical topics vs. practical ones, insufficient training courses, cross-sectional training, selective presence of volunteers, and not supervising them in the application of the knowledge they have gained in the training sessions were among the challenges identified by the stakeholders:

“*The empowerment classes were mainly focused on getting volunteers familiar with theoretical definitions. You can find these contents just by Googling. These courses will not add to my skills. I really need technical expertise not theoretical issues. What is the use of knowing the theoretical definitions?...I do not know how to define a group but have joined it....” (a volunteer, empowerment workshop)*

One of the main challenges most volunteers mentioned was insufficient support from the municipality:

“*Last year, on the 4th of December, the volunteer day, we were invited to a ceremony. Only one of us was invited to present herself and was prized in the ceremony. We are not obsessed to be seen, but there are places where you need to feel you and your activities are counted. I am still interested in volunteering. I am now in charge of public relations for the children of Shush districts, but there are times we need appreciation”(A* volunteer, focus group*)*

Insufficient human capacities was another challenge most stakeholders stated:

“*We have contacted community nutritionist of the health centers in the MOHME, they have defined roles to communicate with their neighborhood and have educational classes outside the health centers. Some participated for educational classes for members of the obesity clubs in health houses in some districts; however, they do it without any pre-planning and/or monitoring. They just take it for granted.” (A volunteer, interview)*

Low funding for health promotion activities was one of the challenges most volunteers stated:

“*We do not have the budget to hold leisure tours. In the time of Dr. … management of the municipality, we were paid for renting bus and related facilities to hold leisure and sport tours. This has been cut off for years now. Managers say we do not have budget and this has limited our activities.” (local manager, focus group)*

Another challenge identified was the weakness of the exercise and sports activities of the clubs. Some neighborhoods had physical or cultural limitations for public exercise; however, some efforts had been made by volunteers to bargain for facilitating these activities.

Since the COVID-19 pandemic, most activities in obesity clubs are based on virtual activities, e.g., virtual nutrition education sessions. However, there are challenges in this regard due to infodemic, and poor management and control over the content of messages, to assure scientifically sound and evidence based:

“*Volunteer leaders mainly forward health messages from other groups they receive, and in some cases, the content is not scientifically sound with no truth checking.” (local manager, interview)*

Other challenges identified included poor collaboration or motivations of volunteers due to factors such as low support or personal problems, disentitling problems, low food and nutrition literacy, instability of the programs, and poor participatory documentation and evaluation.

### Suggestions for better performance and community participation of the obesity prevention clubs

#### Suggestions from the focus group discussion session

Volunteer leaders' suggestions and their ranks to improve the function of obesity clubs in obesity prevention programs and improve participation are presented in Table 3. Designing valid ID cards for volunteers to facilitate their entry into public organizations ranked the highest score.

**Table 3:** Stakeholder's suggestions and their ranking regarding improvement of the function of municipality health clubs in obesity prevention and improving participation (based on the focus group discussion results).

**Suggestions to improve the program**	**Rank**
Designing valid ID card for volunteers to facilitate their entry in public organizations	36
Team-building with a selected coach and verifying the nutritional training content provided by the trainers	35
Dedicating a day to obesity prevention clubs and their volunteers in the country calendar	34
Accompanying the volunteers with the officials of organizations in activities related to food and physical activity environment at neighborhood level	34
Improving the quality of human resources participating in trainings	33
Establish a rewarding mechanism for standard weight loss for obesity prevention club members	33
Providing happy and friendly meetings with a focus on healthy eating and physical activity	30
Expanding virtual activities and advertisements of clubs	29
Forming NGOs related to obesity prevention	23

#### Suggestions for the future direction

The main themes were extracted based on the content analysis of data collected from focus group discussions, interviews, and related documents, and considering the point of view of different stakeholders involved are listed in Table 4.

**Table 4:** Suggestions to improve function of health clubs in obesity prevention programs.

	**Themes (*n* = 17)**	**Sub-themes (*n* = 57)**
1	Continuing and purposeful training and empowerment of volunteers of the obesity prevention clubs for participatory planning	• Constant presence of facilitator in the structure of the obesity clubs for training and empowering the volunteers (2) • Empowerment and training of volunteers in problem identification and need assessment (1, 2) • Improving volunteers' skills in identifying local resources (human and financials) (1, 2) • Improving advocacy skills of volunteers (2) • Improving faciliatory skills (2) • Improving documentation and participatory evaluation skill (2)
2	Promotion of independence in the structure and function of the obesity clubs	• Forming NGOs related to obesity prevention (1, 2) • Reduction the dependency of obesity clubs on government municipal bodies (2) • Promotion of participatory down-top planning by volunteers and citizens (2)
3	Community-based and environmental interventions for obesity prevention	• Entering the obesity prevention clubs to local institutions and organizations and going beyond health neighborhood homes for obesity prevention initiatives (1) • Promotion of local health food environment (1)
4	Promoting and facilitating participation of the obesity prevention clubs for advocating changes	• Recognition of citizens and volunteers about health rights related to healthy weight (right to healthy food and healthy food environment) (3) • Recognition of citizens and volunteers about government policies and targets related to obesity prevention (3) • Recognition the right of local CBOs such as obesity prevention clubs by governments and institutions (2) • Changing the attitude of municipal officials to the real participation not manipulation (1) • Creating credit for volunteers to facilitate to enter and act in organizations (1) • Accompanying the volunteers with local or health managers of different organizations in activities related to food and physical activity environment at neighborhood level (1)
5	Expanding cooperation and interaction between the university and obesity clubs	• Promoting the culture of volunteering in the field of community nutrition among students and professors of nutrition schools to cooperate with local obesity prevention clubs (1, 2) • Launching public section in nutrition seminars and interaction of professors and specialists with obesity prevention clubs (1, 2) • Educate nutrition students about community-based research (2) • Involve citizens and CBOs in research (2) • Improving the community-based activities within university and scientific organizations
6	Improving food and nutrition and media literacy of volunteers	• Improving media literacy related to food and nutrition information in social media (1, 3) • Holding nutrition education classes for volunteers to update their nutrition information regarding weight management (1, 3)
7	Supportive programs for citizens and volunteers to maintain motivation	• Provide sport facilities for volunteers to keep them motivated (1) • More financial support for the traveling costs in volunteers (1) • Encouragement of volunteers and citizens to join and act in obesity prevention clubs (1, 3) • Considering sports facilities for active members of obesity prevention clubs (1) • Creating motivational mechanism to reduce weight in neighborhoods (1)
8	Improving citizen knowledge about obesity clubs and their function	• Call for membership and recruitment of citizens and specialists in different organizations for voluntary activities joining obesity clubs (1, 2) • Using the media capacity to inform and attract volunteers about the obesity prevention clubs (1, 2) • Use of local capacities for advertisements about obesity prevention clubs based on neighborhood structure and texture (1, 2) • Using urban spaces to inform about obesity prevention clubs (1, 2)
9	Improving strategies for volunteer identification	• Designing valid ID card for volunteers to facilitate their entry in public organizations (1, 2) • Dedicating a day specialized to obesity prevention clubs and volunteers in calendar (1)
10	Pragmatism and realism in aim of obesity prevention clubs	• Organizing and determining the description of specific and result-oriented services for the centers (2) • Avoidance of slogans and cross-sectional goals and more pragmatism in the centers (1, 2)
11	Improvement documentation and reporting in obesity clubs	Improvement the methods of documenting and reporting in obesity clubs (1,2)
12	Expanding collaboration and communication of obesity prevention clubs with other health CBOs	Communicating and cooperating more with members of other health CBOs (1)
13	Renaming the name of obesity prevention clubs due to the negative effect of the word “obesity”	Renaming the name of obesity prevention clubs to healthy nutrition and lifestyle or weight management (1)
14	Boosting interventions tailored to corona condition	• Sharing sport videos virtually through social media group (1) • Virtualization of centers and advertisements in order to expand it (1) • Considering a place for physical activity and group outdoor placement in accordance with protocols (1)
15	Variety in services of obesity prevention clubs	• Holding a competition in the field of correct weight loss methods in neighborhoods (1) • Establish a reward mechanism for standard weight loss for members (1) • Holding happy and prosperous tours to teach healthy nutrition (1) • Launching the neighborhood radio with the participation and using the capacity of the capable volunteer teachers of the center (1) • Holding happy and friendly meetings with a focus on healthy eating and physical activity (1)
16	Upgrading the financial budget of the centers	• Allocate sufficient and specific budget by the health department to empower and support the centers (1, 2, 3) • Promote the attraction of financial resources from public and donors (1, 2) • Budget upgrades for advertising in clubs (1, 2)
17	Improving the process of selecting nutrition trainers in obesity prevention clubs	• Forming a team to select a coach and verify the nutritional training content provided by the trainers (1) • Improving the quality of human resources participating in training (1)

Parentheses indicated that the subthemes stated by: 1. volunteers and citizens, 2. Local managers, 3. Nutritionists.

Proper targeting, continuous training, and empowerment for participatory planning in volunteers and citizens were among the main themes suggested by different stakeholders. Based on an interviewee:

“*In the first step, our clubs must be empowered for problem identification in the neighborhood. We must train them. Our clubs must learn. What is the problem of obesity directly related to? Economic poverty? Mental health? Violence or anger? Cheap food basket? These are just examples. Once the club understands the problem and its causes, the second step is imitating small projects to plan for solving it. They can either plan themselves or find someone to help them do it. There is no need that volunteers know everything, what is important is to gather relevant people based on the nature of the problem to help them initiate a project. Thus, our clubs should be empowered for problem identification and making connections to help them do sth. We have failed in education and empowerment. It's very important to work on it.” (local manager, interview)*

Promoting independence in the structure and function of the obesity clubs was also suggested by stakeholders. A local manager said:

“*The municipal health department has actually worked on the structure of the centers as a governmental or semi-governmental section not a civil society. For example, the health department says I want to do an activity for Diabetes Prevention Week and asked volunteers to do that. They took photos and it is over. Volunteers are waiting for a call from the health department to do sth. if you have not had a plan for this center for a year, volunteers asked why don't you have a plan for us for a year. Do you get? When the center tells you why do you have nothing to do with me? It means it's yours. Its governmental. But if you grow the clubs in a democratized way, the clubs have its own plan for a year and one of the people it took a meeting with, is you, our clubs should be more independent; they should be trained for that.” (local manager, interview)*

“*These clubs should not wait for the programs form the top. They should help the municipality not being behind it. CBO organizations are lunched when we have weaknesses in government action on health.” (local manager, interview)*

Entering the OBCs at local institutions and going beyond health neotheropod homes for obesity prevention initiatives is suggested by some volunteers and local managers:

“*The obesity prevention clubs should move beyond the neighborhood health house and connect to different organizations in their neighborhood to initiate prevention activities. An employee seldom goes to neighborhood health house. She or he doesn't have the time to use facilities offered by health houses. We are wrong to wait for citizens to come and get involved without introducing ourselves to them. If they know us, they may follow us. For example, we should go to workplaces and organizations and advocate changes. We can convince managers to make conditional promotions for the employees who have normal weight.” (Volunteer, focus group)*

“*We have to go to organizational environment such as schools. Schools buy unhealthy foods. We can initiate small projects such as healthy snacks and breakfast for them.” (Volunteer, interview)*

Familiarizing citizens and volunteers about health rights related to healthy weights (right to healthy food and healthy food environment) are among the suggestions stakeholders stated:

“*…if we want to move from obesity treatment approach to disease prevention and act more effectively, I think the citizens and volunteers should be more aware of their own health rights. I think we have rights in regards to healthy nutrition too. Citizens have the right to eat healthy food or make sure that there is healthy oil in their processed food. Parents have the right that schools serve healthy foods to their children. Do volunteers know what are the government programs and targets to prevent obesity? They have to be aware of that. When they become informed about their rights, they will ask for it. Real participation in health starts with knowing more about your rights and then demand for it. (Nutritionist, interview)”*

## Discussion

The results of this study showed that, based on the stakeholders' views, despite the activities of OBCs in engaging citizens to improve physical activity and eating behaviors at the neighborhood level, several challenges hinder their effectiveness. A poor marketing strategy was identified as a major weakness of OBCs, as only a few neighbors were informed about them. The stakeholders recommended several strategies in this regard, including calling for membership in related organizations to attract volunteers to the clubs, using the capacity of the media, and urban advertising.

Although annual training workshops were conducted to empower volunteers' skills and improve their capacity in participatory planning in the OBCs, these workshops were not practical and skill-based, and as a result, volunteers were still weak in applying these skills. Empowerment is a long-term process, and therefore investment in it will require skill-based training and further follow-up to run small neighborhood participatory projects. Belzian et al. analyzed the experiences of the projects implemented within the Healthy Municipalities and Communities Program (HMCP) for promoting healthy eating and physical activity in Argentina and reported similar concerns regarding training on project design, funding, and political support from local authorities (30). Poor participatory planning skills of volunteers are an important factor that can affect their effectiveness.

Low motivation and organizational support for volunteers were the next challenges stated regarding OBCs. Similar findings have been reported in other volunteer-based studies in Iran, e.g., the healthy volunteer program under the supervision of MoHME (30, 31). Transforming the obesity prevention clubs into NGOs to assure more independence was among the perceived suggestions proposed by the study participants. This is in line with the research by Damari et al. (32) who evaluated women's health volunteers' program in Iran. Likewise, they suggested managing the women's health volunteer program as a non-governmental organization (NGO) under the supervision of MoHME (32).

Another challenge raised was the poor management of nutrition information shared through OBC-related virtual groups in social media, as well as nutrition education offered by traditional medicine-based teachers. Increasing trust and community-academia partnership through formal and informal communication with the local community is needed to ensure sound nutrition education in the community (33).

In the meantime, the need for advocacy for changing the socio-ecological determinants of obesity, i.e., the food environment in neighborhoods was suggested by the stakeholders. Applying citizen science approaches for monitoring enabling/disabling food and physical neighborhood environment, as well as using photovoice projects to explore environmental determinants of obesity, can enhance local activities on socio-ecological determinants are warranted. The need for an enabling environment to support participants and facilitate local community-based organizations or NGOs in Iran, has also been emphasized by Damari et al. (34). Introducing new methods for participatory planning in training classes, including using citizen science approaches for monitoring enabling/disabling food and the physical neighborhood environment as well as using photovoice projects to explore environmental determinants of obesity can enhance local activities on socio-ecological determinants.

Poor documentation and evaluation capacities were other identified challenges. Local managers were only obliged to report activities they did without training about a community-friendly but scientific approach to understanding the effects of the program. Other studies in different contexts (30, 35) and in Iran (32) have found that the evaluation of local projects was mostly based on registering the fulfillment of proposed activities rather than process and outcome indicators (30). Improving evaluation capacity, especially through participatory approaches, for stakeholders involved in health promotion programs should be promoted. Expanding organizational evaluation culture and not seeing evaluation as a chore to meet reporting obligations are recommended. Improving the capacities of the metacognition and reflection sessions such as Gibbs model of reflection (36, 37) within community members and local managers are warranted. The replacement of participatory evaluation courses with non-practical ones during in-service training for local managers could help.

The constant presence of facilitators in the structure of the obesity clubs for training and empowering the volunteers was one of the main suggestions of the stakeholders. This is in line with previous studies that emphasized the importance of recruiting facilitators and community advisors to work with volunteers in order to enhance community participation in health promotion programs (38, 39). In addition, similarly, Mohamadi et al. reported that using appropriate channels of communication in accordance with the culture of the community, providing economic incentives, and creating a pro-participation environment are the most important ways to attract community participation (39). Investment in the literacy and skills of volunteers for health activities within the obesity prevention clubs reemphasizes the findings of previous studies. For example, Vareilles et al. showed that health volunteers should be supervised and supported *via* formal training (40). Middleton et al.'s study, which aimed to explore the experiences of implementation of a community-based obesity prevention program by stakeholders, revealed similar results to this study. The stakeholders mentioned that more time and resources must be provided to establish a long-lasting working partnership and effective coalitions between the multi-agency organizations involved in the community-based obesity program(s) (41). Similar to our study, they also highlighted the importance of communication and marketing issues and added participatory approaches to traditional evaluation in the processes of planning, implementation, and evaluation of a community-based obesity prevention program (41). The evidence on community-based initiatives for obesity prevention has emphasized the importance of using proper frameworks and theories for behavioral change in program planning (42); however, in developing the obesity prevention clubs, no framework or theory has been used.

Several considerations need to be taken into account in developing community-based obesity prevention programs. King et al. highlighted a number of best practices to consider in this regard, including governance and transparency, implementation and sustainability, evaluation, program design and planning, and community engagement (43). The World Obesity Federation also identified 10 evidence-based considerations to support the successful implementation of community-level obesity prevention (42): (1) undertake a situation analysis of the current health situation, (2) consider budget, (3) use frameworks and theories, (4) adopt an integrated approach, (5) implement several and multi-component interventions, (6) engage stakeholders, but safeguard processes from conflict of interests, (7) ensure political engagement, (8) set benchmark indicators, (9) seek community groups' participatory mechanisms, and (10) ensure that interventions are sustainable.

### Lessons learned

This study evaluated a community-based program, i.e., OBCs in Tehran city through participatory research (PR) and a community-based participatory evaluation approach. Despite the importance of PR as a viable approach to academic and community engagement, it is frequently overlooked in research paradigms (19) due to several factors in different contexts (44). In Iran, Bahraminejad et al. identified four factors as challenges that influence community-based participatory research: (1) “interpersonal relationships” needed for mutual collaboration between community and professional stakeholders, including communication, trust, and respect, (2) “readiness”, (3) “environment-conducive”, and (4) “institutional issues such as intuitional value system” (45).

The application of the participatory approach to evaluation in this study is one of its main strengths, as it provided an interactive learning environment. However, based on reflections during the evaluation process, the findings need to be interpreted with caution, as participatory evaluation is time-consuming and requires sufficient time and a long-term partnership. Making evidence to act also needs long-term university-community partnerships. In addition, relevant literature has shown that implementing a participatory approach to evaluation will require trust and mutual collaboration between the university and the community. This can be a challenge, and time and skill are needed to build mutual vision and trust. Although we tried to manage it somehow through joining these clubs and ceremonies, it was quite time-consuming for a higher education academician but rewarding. Furthermore, like other participatory evaluation studies, the present findings are mostly focused on the subjective judgment of the stakeholders involved. Building evaluation capacity of community members (46), gaining community trust, having good facilitation skills, dedicating sufficient time for dissemination of the findings (47), constant and open dialogue among partners during the implementation of the participatory evaluation (48), and having flexibility (48) to make changes in the evaluation plan based on context and characteristics of the programs, are all recognized as critical steps when applying the participatory approach to evaluation.

Based on the findings, we can conclude that there are gaps and weaknesses in all stages of the WHO framework on community participation, including informing, consulting, collaboration, and empowerment (6), in these OBCs. We offer some policy implications that might help improve the capacity of OBCs in the future. Strengthening community coalitions for obesity prevention at the local level is strongly warranted. The municipal health department needs to develop strategies to facilitate more favorite environments to involve potential governmental sectors, academia, and community members for obesity prevention at neighborhood levels. Moreover, more focus is needed on long-term investment and funding for empowerment and supporting volunteers for making better network and participatory planning to identify their needs and priorities and run community-based programs tailored to their needs for obesity prevention at the neighborhood level.

We also suggest community nutrition specialists and academia to enhance their efforts toward improving community-academia partnerships. In addition, the nutrition academia needs to institutionalize CBPR and invest in more training about how to do CBPR research and translate knowledge to put participatory research data into practice. Revisions in the educational curriculum can be a step in this regard. The academia also needs to improve its trust and networking opportunities with the local community to empower citizens about obesity prevention.

## Conclusion

This study can enrich the current understanding regarding local community-based initiatives in the non-health sector and participatory evaluation practice in Iran and can serve as a case study in a middle-income country. Strengthening community coalitions for obesity prevention at the local level is strongly warranted. Organizational support and training about participatory planning for health volunteers and strategies to strengthen community coalitions for obesity prevention at the local level need to be strengthened. The municipality should facilitate a more enabling environment and improve governance by involving citizens, neighborhood social capital, health volunteers, academia, and all potential governmental sectors within and outside the municipality to collaborate for obesity prevention.

## Data availability statement

The original contributions presented in the study are included in the article/Supplementary material, further inquiries can be directed to the corresponding author.

## Ethics statement

The studies involving human participants were reviewed and approved by Research Ethics Committees of National Nutrition and Food Technology Research Institute IR.SBMU.NNFTRI.REC.1398.071. The patients/participants provided their written informed consent to participate in this study.

## Author contributions

SE was involved in the conceptualization of the study, data collection, data interpretation, and writing of the initial manuscript draft. NO and AT supervised the project and were involved in the conceptualization and the study design. RM was involved in guiding the project. DG was involved in guiding the project and involved in the thematic analysis of qualitative data (as the second coder). FR was involved in making the evaluation team and protocol as well as facilitating the participatory evaluation process. All authors contributed to the manuscript's revision and finalization. All authors contributed to the article and approved the submitted version.

## References

[1] Ataey A Jafarvand E Adham D Moradi-Asl E. The relationship between obesity, overweight, and the human development index in World Health Organization Eastern Mediterranean Region Countries. J Prev Med Public Health. (2020) 53:98. 10.3961/jpmph.19.100 32268464 PMC7142010

[2] Motlagh ME Ziaodini H Qorbani M Taheri M Aminaei T Goodarzi A. Methodology and early findings of the fifth survey of childhood and adolescence surveillance and prevention of adult noncommunicable disease: the Caspian-V Study. Int J Prev Med. (2017) 8. 10.4103/2008-7802.198915 28217266 PMC5288959

[3] Azadnajafabad S Mohammadi E Aminorroaya A Fattahi N Rezaei S Haghshenas R. Non-communicable diseases' risk factors in Iran; a review of the present status and action plans. J Diabetes Metab Disord. (2021) 1–9. 10.1007/s40200-020-00709-8 33500879 PMC7821170

[4] World Health Organization. Global Action Plan for the Preven-Tion and Control of Ncds 2013-2020. (2013). Available online at: https://www.who.int/nmh/publications/ncd-action-plan/en/ (accessed February 12, 2020).

[5] Amerzadeh M Salavati S Takian A Namaki S Asadi-Lari M Delpisheh A. Proactive Agenda Setting in Creation and Approval of National Action Plan for Prevention and Control of Non-Communicable Diseases in Iran: the use of multiple streams model. J Diabetes Metab Disord. (2020) 1–12. 10.1007/s40200-020-00591-4 PMC11599664 39610473

[6] World Health Organization. Community Participation in Local Health and Sustainable Development: Approaches and Techniques. (2002). Available online at: https://apps.who.int/iris/handle/10665/107341 (accessed Feburary 6, 2023).

[7] Rifkin SB. Lessons from community participation in health programmes: a review of the post alma-ata experience. Int Health. (2009) 1:31–6. 10.1016/j.inhe.2009.02.001 24036293

[8] Ardakani MA Rizwan H. Community ownership and intersectoral action for health as key principles for achieving “Health for All”. East Med Health J. (2008) 14(Suppl.):S57–66. Available online at: https://apps.who.int/iris/bitstream/handle/10665/117586/14_s1_s57.pdf (accessed Feburary 6, 2023). 19205604

[9] World Health Organization. Concepts and Methods of Community-Based Initiatives. (2003). Available online at: https://apps.who.int/iris/handle/10665/116357 (accessed Feburary 6, 2023).

[10] Sein UT. Health volunteers: third workforce for health-for-all movement. In: Regional Health Forum, Vol. 10. World Health Organization (2006). p. 38–48. Available online at: https://apps.who.int/iris/bitstream/handle/10665/205773/B0234.pdf?sequence=1#page=43

[11] World Health Organization. Address by Dhag, Regional Director, WHO Eastern Mediterranean Region, to the Intercountry Meeting to Finalize the Training Manual for Community Representatives and Health Volunteers on Priority Health-Related Programmes, Cairo, Egypt. (2006). Available online at: https://apps.who.int/iris/handle/10665/124689 (accessed Feburary 6, 2023).

[12] World Health Organization. Intercountry Meeting to Review the Current Status of Community-Based Initiatives Programmes and Establish Future Directions and Actions. Report of an Intercountry Meeting, Cairo, Egypt. (No. WHO-EM/CBI/062/E) (2009). Available online at: https://apps.who.int/iris/handle/10665/115964 (accessed Feburary 6, 2023).

[13] Eftekhari MB Mirabzadeh A Forouzan AS Dejman M Afzali HM Djalalinia S. A qualitative study of community-based health programs in iran: an experience of participation in Ir Iran. Int J Prev Med. (2014) 5:679. 10.5539/gjhs.v5n3p28 25013686 PMC4085919

[14] Hsu J Majdzadeh R Harichi I Soucat A. Health System Transformation in the Islamic Republic of Iran: An Assessment of Key Health Financing Governance Issues. World Health Organization (2020). Available online at: https://apps.who.int/iris/bitstream/handle/10665/333760/9789240003774-eng.pdf (accessed Feburary 6, 2023).

[15] Municipal Health Department. Executive Guideline of Health Programs in Municipal Health Department. Department of Social and Cultural affairs of Tehran Municipality, Health Department, Tehran, Iran. (2018).

[16] Minkler M Wallerstein N. Community-Based Participatory Research for Health: From Process to Outcomes. John Wiley & Sons (2011).

[17] Cargo M Mercer SL. The value and challenges of participatory research: strengthening its practice. Annu Rev Public Health. (2008) 29:325–50. 10.1146/annurev.publhealth.29.091307.083824 18173388

[18] Jolley G. Evaluating complex community-based health promotion: addressing the challenges. Eval Progr Plann. (2014) 45:71–81. 10.1016/j.evalprogplan.2014.03.006 24755377

[19] Washburn LT Traywick L Thornton L Vincent J Brown T. Using ripple effects mapping to evaluate a community-based health program: perspectives of program implementers. Health Promot Pract. (2020) 21:601–10. 10.1177/1524839918804506 30366499

[20] Richardson L. Participatory evaluation. In: Handbook of Social Policy Evaluation. Edward Elgar Publishing (2017). p. 119–40.

[21] Nitsch M Waldherr K Denk E Griebler U Marent B Forster R. Participation by different stakeholders in participatory evaluation of health promotion: a literature review. Eval Progr Plann. (2013) 40:42–54. 10.1016/j.evalprogplan.2013.04.006 23732440

[22] Chevalier JM Buckles DJ. Handbook for Participatory Action Research, Planning and Evaluation. Ottawa: Sas2 Dialogue (2013). p. 155.

[23] UNICEF. Games and Exercises: A Manual for Facilitators and Trainers Involved in Participatory Group Events. Kenya: UNICEF-Esaro, Communication Section; New York, NY: UNICEF (2004).

[24] Crishna B. Participatory Evaluation (I)–sharing lessons from fieldwork in Asia. Child Care Health Dev. (2007) 33:217–23. 10.1111/j.1365-2214.2006.00657.x 17439432

[25] Crishna B. Participatory evaluation (Ii)–translating concepts of reliability and validity in fieldwork. Child Care Health Dev. (2007) 33:224–9. 10.1111/j.1365-2214.2006.00658.x 17439433

[26] Barghouti Z. Participatory Evaluation: Theories + Methods for Remote Work. (2020). Available online at: https://evalparticipativa.net/wp-content/uploads/2021/06/31.-participatory-evaluation_-theories-methods-for-remote-work.pdf (accessed Feburary 6, 2023).

[27] Mckee N Solas M Tillmann H. Games and Exercises–a Manual for Facilitators and Trainers Involved in Participatory Group Events. Nairobi: UNICEF-ESARO (1998).

[28] Tong A Sainsbury P Craig J. Consolidated criteria for reporting qualitative research (COREQ): a 32-item checklist for interviews and focus groups. Int J Qual Health Care. (2007) 19:349–57. 10.1093/intqhc/mzm042 17872937

[29] Hall R Brent Z Franco J Isaacs M Shegro T. Toolkit for Participatory Action Research. International Development Research Centre (IDRC) (2017).

[30] Belizan M. Chaparro Rm, Santero M, Elorriaga N, Kartschmit N, Rubinstein Al, et al. Barriers and facilitators for the implementation and evaluation of community-based interventions to promote physical activity and healthy diet: a mixed methods study in Argentina. Int J Environ Res Public Health. (2019) 16:213. 10.3390/ijerph16020213 30646502 PMC6352269

[31] Moghaddam Hr Allahverdipour H Matlabi H. Successful recruitment and retention strategies for women health volunteers: viewpoints of the volunteers' supervisors and relevant researchers. J Multidiscip Healthcare. (2018) 11:621. 10.2147/JMDH.S180544 30464495 PMC6208547

[32] Damari B Riazi-Isfahani S. Evaluating the women health volunteers program in Iran-a quarter century experience (1992–2016). Arch Iran Med. (2018) 21:566–71. 30634853

[33] Al Jawaldeh A Edalati S Omidvar N. Covid-19 pandemic and needs to invest in improving public media food and nutrition literacy. Nutr Food Sci Res. (2021) 8:1–3.

[34] Damari B Heidarnia M Rahbari B. Role and performance of Iranian Ngos in community health promotion. Payesh. (2014) 13:541–50.

[35] Rice M Franceschini M. Lessons learned from the application of a participatory evaluation methodology to healthy municipalities, cities and communities initiatives in selected countries of the Americas. Promot Educ. (2007) 14:68–73. 10.1177/10253823070140021501 17665701

[36] Asselin ME Fain JA. Effect of reflective practice education on self-reflection, insight, and reflective thinking among experienced nurses: a pilot study. J Nurses Prof Dev. (2013) 29:111–9. 10.1097/NND.0b013e318291c0cc 23703269

[37] Vizeshfar F Momennasab M Yektatalab S Iman M. Empowering health volunteer's through participatory action research in a comprehensive healthcare center. BMC Public Health. (2021) 21:889. 10.1186/s12889-021-10878-7 33971852 PMC8112012

[38] Onyango G Worthen M. Handbook on Participatory Methods for Community-Based Projects: A Guide for Programmers and Implementers Based on the Participatory Action Research Project with Young Mothers and their Children in Liberia, Sierra Leone, and Northern Uganda PAR. University of Wyoming (2010).

[39] Mohamadi NK Bahreini F. A review on the role of community participation in health promotion programs. Depict Health. (2019) 10:310–8.26745867

[40] Vareilles G Pommier J Kane S Pictet G Marchal B. Understanding the motivation and performance of community health volunteers involved in the delivery of health programmes in Kampala, Uganda: a realist evaluation protocol. BMJ Open. (2015) 5:e006752. 10.1136/bmjopen-2014-006752 25631314 PMC4316434

[41] Middleton G Henderson H Evans D. Implementing a community-based obesity prevention programme: experiences of stakeholders in the North East of England. Health Promot Int. (2014) 29:201–11. 10.1093/heapro/das072 23297339

[42] World Obesity. Community-Level Interventions to Address Obesity: Considerations for European Policymaker. Available online at: https://s3-eu-west-1.amazonaws.com/wof-files/communitylevel_interventions_to_tackle_obesity_-_considerations_for_european_policymakers_v9.pdf (accessed Feburary 6, 2023).

[43] King L Gill T Allender S Swinburn B. Best practice principles for community-based obesity prevention: development, content and application. Obes Rev. (2011) 12:329–38. 10.1111/j.1467-789X.2010.00798.x 20880111

[44] Baum F. Challenges in community-based participatory research. Med J Aust. (2018) 209:489. 10.5694/mja18.01014 30521442

[45] Bahraminejad N Majdzadeh R Haliza R Faisal I Azimi H. Factors affecting community-based participatory research in iran: a qualitative study on health sector stakeholders' view. J Res Health. (2018) 8:68–78. 10.29252/acadpub.jrh.8.1.68

[46] Srivastava A. Challenges for evaluation practices and innovative approaches: lessons during Covid-19 pandemic. Eval Progr Plann. (2022) 92:102095. 10.1016/j.evalprogplan.2022.102095 35500477 PMC9020492

[47] Salabarría-Peña Y Robinson WT. Going beyond performance measures in HIV-prevention: a funder-recipient expedition. Eval Progr Plann. (2022) 90:101996. 10.1016/j.evalprogplan.2021.101996 34507834

[48] Scarinci IC Johnson RE Hardy C Marron J Partridge EE. Planning and implementation of a participatory evaluation strategy: a viable approach in the evaluation of community-based participatory programs addressing cancer disparities. Eval Progr Plann. (2009) 32:221–8. 10.1016/j.evalprogplan.2009.01.001 19232727 PMC2833106

