# Supplementary material for: Participatory evaluation of municipal obesity prevention clubs in Tehran city: Strengths, challenges, and future direction
Source: Front Public Health. 2023 Feb 16;11:1055210. doi: 10.3389/fpubh.2023.1055210 (PMC9978347; doi:10.3389/fpubh.2023.1055210)
Supplement: Supplementary file 1 [file Data_Sheet_1.docx]

Supplementary file 1

Supplementary table 1-List of documents analyzed for evaluating obesity prevention clubs

| Type of documents | Title | Number (97) |
| --- | --- | --- |
| Development plans of Tehran (in Persian) | 2rd 5-year plan for the development of Tehran city (2015-2019) | 2 |
|  | 3rd 5-year plan for the development of Tehran city (2020-2024) |  |
| Executive guidelines related to community based Health/Obesity prevention clubs (in Persian) | Statue law program of Obesity prevention clubs (2014) | 9 |
|  | Executive manual and guidelines of Health community-based (CB) health organizations (clubs) (2014-2015) |  |
|  | Executive manual of health department in 22 districts (2016) |  |
|  | Executive manual of health department (2016) |  |
|  | Obesity prevention league (2016) |  |
|  | Executive manual of municipal health department (2017) |  |
|  | Executive guideline of health programs in municipal health department (2018) |  |
|  | Executive guideline of health programs in municipal health department (2019) |  |
|  | Executive guideline of health programs in municipal health department (2020) |  |
| Reports and studies related to health activities of Tehran municipality (in Persian) | Overweight and obesity conference of interventions related to URBAAN health project (2015) | 8 |
|  | Obesity prevention reports of urban HEART project (2015) in different districts in Tehran |  |
|  | Twelve-year report of the activities of the Health Department of Tehran Municipality (2006-2018) |  |
|  | Citizen survey report of activities of the General Health Department of the municipality of Tehran (2017) |  |
|  | Analytical report of the summer school of community-based health clubs (2019) |  |
|  | The knowledge and lived experiences of the health family of Tehran municipality (2019) |  |
|  | Performance report of the National Health Week of the Municipal Health Department (2020) |  |
|  | The detailed report of the of municipal social and cultural affairs (2020) |  |
| Minutes of the meetings of obesity prevention clubs (in Persian) | Minutes of the meetings of the regional volunteers of the obesity prevention clubs (2014-2020) | 40 |
| Books (in Persian) | Assessment of obesity clubs (2013) | 4 |
|  | Urban management and health) (2014) |  |
|  | First experience of interventions in urban HEART project in Tehran city (2017) |  |
|  | Future path of municipal health houses (2017) |  |
| News related to obesity prevention clubs and obesity prevention interventions in urban HEART program in formal national news-agencies (in Persian) | Published News regarding activities within obesity prevention clubs in national formal news-agencies (2014-2019) | 23 |
|  | Published News regarding obesity prevention interventions in urban HEART program (2015-2017) | 8 |
| Related reports and studies | Public voice and participatory governance in the health sector: status quo and way forward (49) | 2 |
|  | Report on documentation and evaluation of Urban HEART pilot in Tehran, Islamic Republic of Iran (50) |  |
| Related articles | Effects of Community-Based Interventions in Reducing the Obesity and Overweight Rate Among Iranian Women: Using Urban Health Equity Assessment and response tool (51) | 1 |

**References:**

49. Rajan D, Hadi Ayazi M, Moradi-Lakeh M, Rosami-Gooran N, Rahbari M, Damari B. *Chapter 4: Public Voice and Participatory Governance in the Health Sector: Status Quo and Way Forward. Health System*

*Transformation in the Islamic Republic of Iran: An Assessment of Key Health Financing and Governance Issues*. Geneva: World Health Organization (WHO) (2020).

50. Allahverdipoor H, Behdjat H, Tajaddini N, Vahidi RG, Jabbari H. *Report on Documentation and Evaluation of Urban Heart Pilot in Tehran, Islamic Republic of Iran*. Tabriz: National Public Health Management Centre, Tabriz Univ Med Sci (2013).

51. Rassam F, Khedmat L, Khatami F. Effects of community-based interventions in reducing the obesity and overweight rate among Iranian women using urban health equity assessment and response tool*. Int J Travel Med Global Health*. (2019) 7:99–104. doi: 10.15171/ijtmgh.2019.21

**Supplementary file 2 -Semi-structured Interview guide**

-Tell me about yourself/ your organization/ what is the story of your participation/volunteering/membership in OBCs? When and why have you joined it, how long is it, and what activities have you been involved in? What is your specific role in this regard?

-What do you know about the history of OBCs formation and its related factors? Please explain it

-What are the goals of OBCs, Do you think the programs within OBCs have been successful to achieve these goals? Please explain it

-What services do OBCs provide in general/your district, neighberhood? Do you think these services meet citizens’ needs in your district/neighborhood? What is your opinion on the quality OBCs services?

-Which organizations and who have you ever collaborated with to initiate obesity prevention activities in OBCs? Can you describe your previous experience in this regard? Based on your experience, what challenges have you faced in this regard?

-How do you engage community and citizens in obesity prevention activities in OBCs? Are you successful with them?

-Would you describe the level of participation in OBCs?

-How do you evaluate the physical and human resources in OBCs?

-What is your opinion about the evaluation of activities in OBCs? Has been any report in this regard yet?

-Please explain your general perception of OBCs. How do you assess the role of yourself/ other stakeholders involved in obesity prevention in OBCs? Are you/they successful to gain aims in your district/neighborhood?

-Based on your experience, what were the strengths, issues/challenges or barriers that you and OBCs encountered over the past years? Which aspects of the programs need to be changed or improved? Please describe in details.

-What suggestions do you have to overcome these barriers or for improvement of OBCs?

-Are there other people or key informants you think we should talk/interview?

-Thank you for participating in this interview. Do you have any other ideas or comments related to this topic you would like to add?

Note: The questions were adjusted based on each interviewee’s position/role in OBCs”
